# A Rare Case of Linear Phlebolith: Foreign Body Discovered in the Femoral Vein

**DOI:** 10.1155/cris/8824786

**Published:** 2025-01-10

**Authors:** Tariq Alanezi, Abdulmajeed Altoijry, Kaisor Iqbal, Saeed Alabduljabbar, Mohammed Yousef Aldossary, Sultan AlSheikh

**Affiliations:** ^1^Division of Vascular Surgery, Department of Surgery, College of Medicine, King Saud University, Riyadh 11322, Saudi Arabia; ^2^Division of Vascular Surgery, Department of Surgery, Eastern Health Cluster, Qatif Central Hospital 32654, Qatif, Saudi Arabia; ^3^Division of Vascular Surgery, Department of Surgery, Dammam Medical Complex, Dammam 32245, Saudi Arabia

**Keywords:** case report, deep vein thrombosis, femoral veins, lower extremity, phlebolith

## Abstract

**Introduction:** Phlebolith is a term that refers to round-shaped calcified thrombi commonly located in the pelvic region. The occurrence of dense, linear calcifications or phlebolith-like formations within the soft tissues of the lower extremities, particularly in the superficial femoral, greater saphenous, or popliteal veins, is rare.

**Patient Concerns:** This study presents the case of a 73-year-old woman who was being evaluated for postmenopausal bleeding. During the patient's diagnostic workup, an incidental linear-shaped phlebolith was discovered. She had a positive history of deep vein thrombosis (DVT) for 36 years following her previous vaginal delivery.

**Diagnosis:** Upon further examination and imaging, the patient was found to have a chronic calcified thrombus in the iliofemoral, popliteal, great saphenous, and superficial femoral veins, which was initially reported as a foreign body in the femoral vein on computed tomography (CT).

**Interventions and Outcomes:** Conservative management was undertaken, with no worsening of her condition upon further follow-up.

**Conclusion:** This study showcased a rare form of a radiographically visible calcified thrombus in the veins of the lower extremities of our patient. Calcified venous thrombosis in the lower extremities is rare, as previously documented cases of venous calcifications have been observed in the pelvis with round shapes or as phleboliths. The common presentations differ from those in our case, making it important to consider such cases when formulating a differential diagnosis. While the precise mechanisms behind the formation of calcified thrombi remain unclear, this study emphasizes the significance of further exploration and future case studies to shed light on this enigmatic phenomenon.

## 1. Introduction

Venous calcification occurs in patients suffering from chronic deep vein thrombosis (DVT) and the resulting post-thrombotic syndrome [1]. The molecular mechanisms that regulate cell-specific pathways leading to calcification remain unclear, although the link to synergistic activation of tumor necrosis factor-alpha with resulting inhibition of transforming growth factor beta that causes smooth muscle calcification has been elucidated [2, 3]. Additionally, involvement of the MAPK-associated pathways [2–6]. Phlebolith is a term that refers to round-shaped calcified thrombi; these thrombi are quite common, mostly found in the pelvic area, and are widely documented in the literature. However, they need to be differentiated from other radiopacities, such as calcified lymph nodes, foreign bodies, sialoliths, and urinary stones [5, 6]. The occurrence of dense, linear calcifications or phlebolith-like formations within the soft tissues of the lower extremities, particularly in the superficial femoral, greater saphenous, or popliteal veins, is rare [5, 6]. In contrast to subcutaneous veins, calcination within the deep veins of the lower extremities following chronic venous thrombosis is uncommon. To date, very few published case studies have explicitly documented this occurrence [1, 3, 4]. In this case report, we describe the clinical presentation of a 73-year-old woman diagnosed with chronic calcified thrombosis affecting the iliofemoral, popliteal, great saphenous, and superficial femoral veins. The patient provided written informed consent for the report of her case details and imaging studies described in this case report.

## 2. Case Presentation

**Figure 1:**
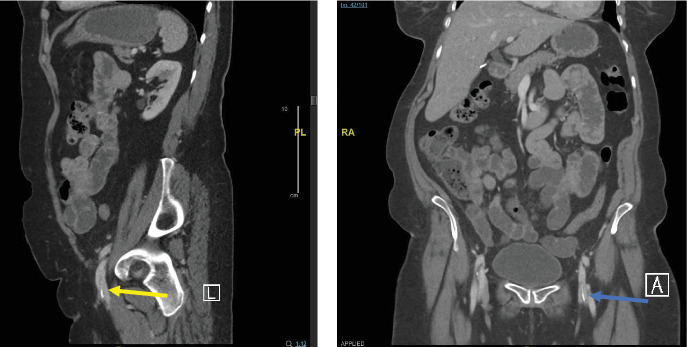
(A) The sagittal computed tomography scan of the abdomen showing a significant-sized linear hyperdense structure in the left common femoral vein (yellow arrow). (B) The coronal computed tomography scan of the abdomen reveals a clear linear hyper-dense structure in the left common femoral vein (blue arrow).

A 73-year-old woman presented to our hospital with a chief complaint of postmenopausal scanty vaginal bleeding. The patient was found to have an incidental endometrial lesion after a comprehensive evaluation by the gynecology department for postmenopausal bleeding. During further assessment of her computed tomography (CT) scan, a significant-sized linear hyperdense structure was found in the left common femoral vein (Figure 1A,B); it was initially thought to be a foreign body, requiring further investigation and assessment.

Her medical history revealed comorbidities, including hypertension, type 2 diabetes mellitus, hyperthyroidism, and dyslipidemia. The patient also reported a significant history of chronic DVT in the left lower limb, which had occurred approximately 36 years prior to her current admission. The patient had no history of gynecological disorders nor any personal or family history of malignancies.

Clinical examination revealed signs of DVT, showing a swollen left lower limb compared to the normal contralateral side. Peripheral pulses were palpable, and varicose veins were visible in the left lower limb.

The laboratory indices of the patient were categorized into general hematology, coagulation, and routine chemistry laboratory values. The coagulation indices were international normalized ratio (0.98), prothrombin time (13.60), and activated partial thromboplastin time (30.30). During routine chemistry, her blood test results were as follows: alanine aminotransferase (18.20), aspartate aminotransferase (17.90), albumin (48.70), alkaline phosphatase (43.00), blood urea nitrogen (3.80), creatinine (56.00), direct bilirubin (1.00), indirect bilirubin (1.50), total bilirubin (2.50), and carbon dioxide (28.30). The general hematology indices included: white blood cells (4.020), red blood cells (4.4), hemoglobin (128.0), hematocrit (39.9), mean corpuscular volume (89.9), mean corpuscular hemoglobin (28.8), mean corpuscular hemoglobin concentration (321.0), red cell distribution width (14.5), and platelet count (250.0).

All laboratory values were within normal limits for the patient; no marker of DVT was found (notably, no rise in D-dimer levels).

**Figure 2:**
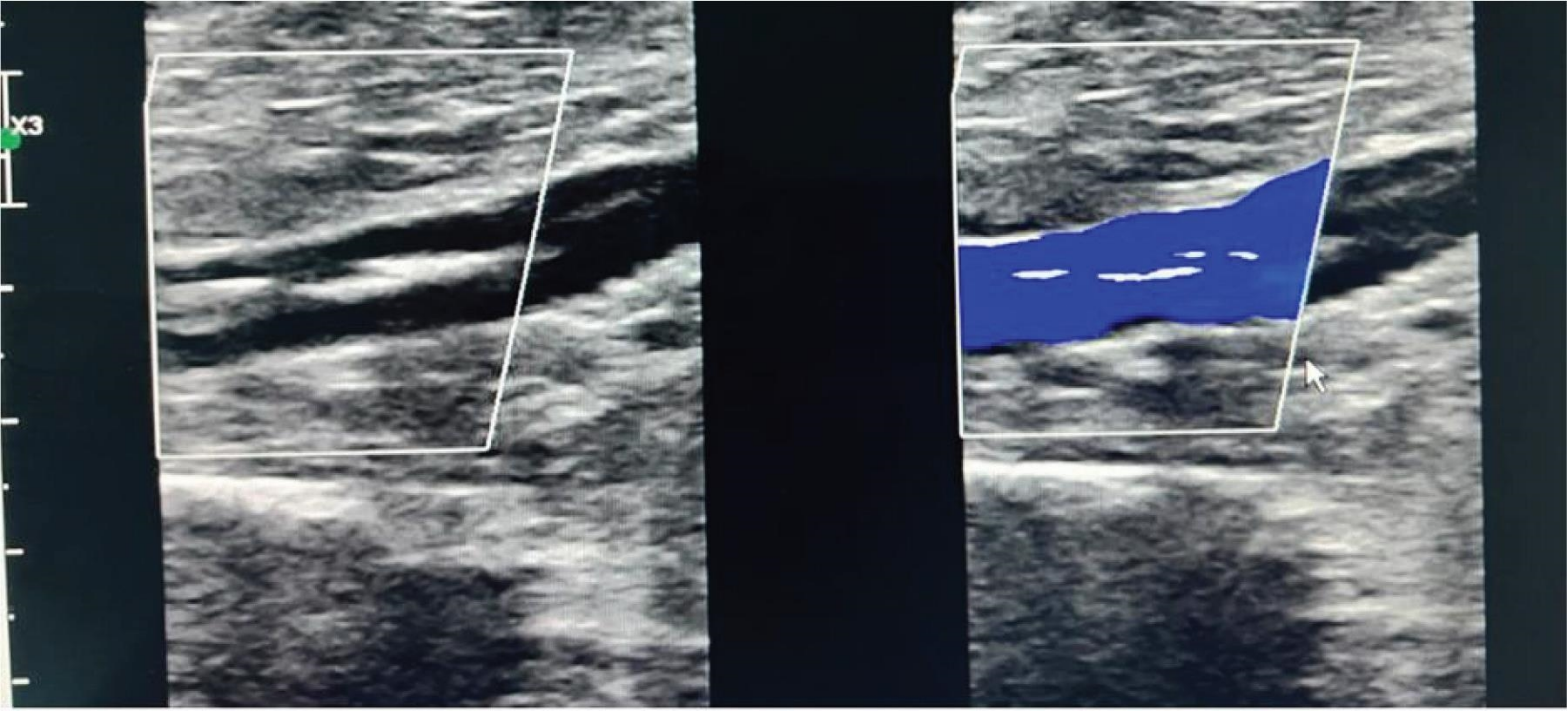
The venous duplex scan of the lower limb showcasing the chronic calcified thrombus with recanalized flow in the popliteal vein.

Lower limb venous duplex revealed a chronic calcified thrombus with recanalized flow in the iliofemoral, popliteal, great saphenous, and superficial femoral veins (Figure 2).

The patient was finally diagnosed with chronic calcified venous thrombosis that involved her iliofemoral, popliteal, great saphenous, and superficial femoral veins. A biopsy of the patient's endometrial lesion also confirmed a diagnosis of a clearly visible, high-grade endometrial carcinoma, specifically of the serous type.

The patient was advised to keep her leg elevated and wear compression stockings as only conservative management was deemed necessary. No additional management was required for the thrombosis. Surgical intervention was undertaken for her endometrial carcinoma via minimally invasive robotic-assisted hysterectomy.

At the 6-month follow-up, the patient was stable, with no worsening or propagation of the thrombus; she did not develop new symptoms or physical findings suggestive of failure of conservative management.

## 3. Discussion

A few relevant cases have been reported since 1975 on the incidence of calcified venous thrombus within the lower extremities [6–11]. The study by Glanz, Park, and Gordon [6] was the first to report the radiographic findings of linear calcifications of venous thrombosis in the popliteal vein, superficial femoral vein, and great saphenous vein. On the other hand, calcifications in the porto-mesenteric venous vasculature have been reported more frequently, especially in patients affected by liver cirrhosis [12]. Indeed, massive calcifications of the venous network have been reported in association with exceptional stress conditions in the portal venous system. This phenomenon has been observed when a porto-caval termino-lateral shunt is combined with a surgical arterial connection between the postshunt ligated main portal vein and a branch of the hepatic artery [13]. Some previous studies have documented the use of bone scintigraphy scans in the detection of DVT in the lower extremities of patients [2, 8, 14]. Zuckier et al. [2] documented the presence of localized Tc-99 m methylene diphosphate (MDP) uptake within the areas affected by DVT using bone scintigraphy. They postulated a potential correlation between this uptake phenomenon and the processes of ossification or calcification occurring within the DVT, a phenomenon sporadically identifiable on radiographic imaging in patients with chronic DVT. A similar study by Sohn et al. [8] reported Tc-99m MDP uptake by multiple phleboliths in the lower extremity. Krmek et al. [9] presented a case of calcified DVT in a female patient with frequent pulmonary sarcoidosis relapses, which is a rare association reported in the medical literature. Banker reported four cases of patients diagnosed with calcified external iliac vein thrombosis [11]. Intraluminal iliac venous calcification was reported by Goodman [10] in a case report of two patients published in 1975. Another study published in 2011 reported the case of a patient with calcified superficial varicose veins who demonstrated spontaneous elimination of white fragments in the lower extremities [7]. A more recent study showed that the proton density-weighted, in-phase stack-of-stars magnetic resonance imaging technique provides calcification visualization in peripheral artery disease [15]. In another case study by Govindu [16] in 2019, radiographs of bilateral lower extremities showcased significant calcifications within the subcutaneous tissue, mostly along the medial aspect of the lower legs. In a recently published case study this year, Guo et al. [17] demonstrated calcification of the lower leg in a patient with chronic lower extremity venous insufficiency and diabetes mellitus.

In this case report, we demonstrate a rare incidence of a chronic calcified venous thrombus in the veins of the lower extremities, including the iliofemoral, popliteal, great saphenous, and superficial femoral veins. Although extremely rare, our findings are consistent with similar reports in previously published studies, and there have been documented case studies confirming the diagnosis of calcified venous thrombosis of the lower extremity [3, 4, 7–10]. Our use of radiographic modalities aligns with previously published case studies that have addressed the detailed detection of calcified venous thrombi in the iliac and lower-extremity veins, which are discernible on radiographs [2, 6–12]. Future research should focus on understanding the underlying pathways that result in the formation of calcified thrombi in the lower extremities. Additionally, different conservative and interventional management strategies to tackle such rare presentations should be explored.

## 4. Conclusion

This case study demonstrated a rare finding: a radiographically visible calcified thrombus in the veins of the lower extremities in a patient with a long history of DVT. To our knowledge, only a limited number of case studies have directly reported findings of calcified thrombi in the deep veins of the lower extremities. The common clinical presentations among the patients in previously documented cases (mostly male patients) were claudication and ulcers of the lower extremities, which contrast with the incidental nature of the diagnosis of calcified venous thrombus in our case. Therefore, physicians should be aware of such cases when considering a differential diagnosis. Thus far, the precise signaling pathways governing the formation of calcified thrombi in lower extremity veins during chronic DVT remain elusive. Further exploration of this phenomenon to better understand the disease process should be the subject of future case studies.

## Data Availability

Data sharing is not applicable to this article as no new data were created or analyzed in this study.

## Nomenclature

DVT: deep vein thrombosis
CT: computed tomography
MDP: methylene diphosphate.
